# Robustness and Information Transfer within IL-6-induced JAK/STAT Signalling

**DOI:** 10.1038/s42003-018-0259-4

**Published:** 2019-01-18

**Authors:** Ulrike Billing, Tomasz Jetka, Lukas Nortmann, Nicole Wundrack, Michal Komorowski, Steffen Waldherr, Fred Schaper, Anna Dittrich

**Affiliations:** 1 Otto-von-Guericke University Magdeburg, Institute of Biology, Department of Systems Biology, Universitätsplatz 2, 39106 Magdeburg, Germany; 2 Polish Academy of Sciences, Institute of Fundamental Technological Research, Division of Modelling in Biology and Medicine, Pawinskiego 5B, 02- 106, Warszawa, Poland; 3 KU Leuven, Department of Chemical Engineering, Celestijnenlaan 200f - box 2424, 3001 Leuven, Belgium

## Abstract

Cellular communication via intracellular signalling pathways is crucial. Expression and activation of signalling proteins is heterogenous between isogenic cells of the same cell-type. However, mechanisms evolved to enable sufficient communication and to ensure cellular functions. We use information theory to clarify mechanisms facilitating IL-6-induced JAK/STAT signalling despite cell-to-cell variability. We show that different mechanisms enabling robustness against variability complement each other. Early STAT3 activation is robust as long as cytokine concentrations are low. Robustness at high cytokine concentrations is ensured by high STAT3 expression or serine phosphorylation. Later the feedback-inhibitor SOCS3 increases robustness. Channel Capacity of JAK/STAT signalling is limited by cell-to-cell variability in STAT3 expression and is affected by the same mechanisms governing robustness. Increasing STAT3 amount increases Channel Capacity and robustness, whereas increasing STAT3 tyrosine phosphorylation reduces robustness but increases Channel Capacity. In summary, we elucidate mechanisms preventing dysregulated signalling by enabling reliable JAK/STAT signalling despite cell-to-cell heterogeneity.

## Abstract (web summary)

Billing et al. clarify mechanisms ensuring reliable cytokine signaling despite variable expression and activation of signaling proteins in cells. Limited activation of signaling proteins either by feedback inhibition or low cytokine doses, and STAT3 serine phosphorylation timely orchestrate robustness and capacity of IL-6-induced signaling.

## Introduction

Life depends on the ability to receive and process information. Information transfer occurs not only between organisms but also between cells within multi-cellular organisms. This cell-to-cell communication is mediated by soluble factors such as cytokines that activate intracellular signalling pathways. Protein copy numbers and activation status of signalling proteins vary strongly even between isogenic cells of one cell type. These variations are caused by extrinsic and intrinsic factors such as fluctuations in the micro-environment, cell-cycle-phase, and stochasticity of protein production, degradation, and activation^1^. At first sight this impedes reliable cellular communication. However, mechanisms evolved to cope with cell-to-cell variability.

The ubiquitous Janus kinase (JAK)/signal transducer and activator of transcription (STAT) pathway orchestrates information transmitted by a large number of cytokines and growth factors, which are involved in the regulation of the immune system, differentiation, growth, and regeneration. In line, dysregulated JAK/STAT signalling is associated with severe developmental, inflammatory, and neoplastic disorders^2^. One of the major activators of JAK/STAT signalling is the cytokine interleukin-6 (IL-6). IL-6 exerts both pro- and anti-inflammatory activities and is e.g. involved in stimulation of B-cells, differentiation of T-cells, and expression of acute-phase proteins in the liver^3^. IL-6 activates a receptor complex consisting of either soluble or transmembrane IL-6 receptor, and the transmembrane glycoprotein 130. Binding of IL-6 to soluble IL-6 receptor induces pro-inflammatory trans-signalling; binding to transmembrane IL-6 receptor induces anti-inflammatory classic signalling. Whereas glycoprotein 130 is expressed ubiquitously, the expression of transmembrane IL-6 receptor is restricted to hepatocytes and leukocytes^4^. However, during inflammation or infection, soluble IL-6 receptor is produced by shedding or alternative splicing, so that virtually all cells respond to trans-signalling^5^. The complex of IL-6 and soluble IL-6 receptor is mimicked by the designer protein Hyper-IL-6 (Hy-IL-6)^6^. The activated IL-6 receptor complex transmits information about the presence of IL-6 from the extracellular space into the cytoplasm. To this end JAKs, which are constitutively associated with glycoprotein 130, become activated. Activated JAKs phosphorylate tyrosine motifs within the cytoplasmic part of glycoprotein 130 that recruit STAT3. Receptor-bound STATs are tyrosine phosphorylated by JAKs, dimerise and translocate into the nucleus where they induce gene expression^7^. In addition to tyrosine phosphorylation STAT3 is phosphorylated at S727. Whereas STAT3-Y705 phosphorylation depends on JAKs, the kinases responsible for IL-6-induced STAT3 serine phosphorylation are less well defined. Activation of protein kinase C (PKC) δ, extracellular signal regulated kinase (ERK)^8,9^, c-Jun N-terminal protein kinase (JNK)^10^, and mechanistic target of rapamycin (mTOR)^11^ results in STAT3-S727 phosphorylation^12^. All these kinases are activated by IL-6^13–15^. IL-6-induced JAK/STAT signalling is terminated by negative regulators such as the feedback-inhibitor suppressor of cytokine signalling 3 (SOCS3) that inhibits JAK activity^16,17^.

IL-6-induced JAK/STAT signalling and its regulation have been studied extensively in cell populations^4,18^. However, analysing cell populations does not consider cell-to-cell heterogeneity and its impact on the reliability of signal transmission. The advent of single cell analyses allows studying mechanisms of cellular signalling in heterogeneous cell populations. To analyse signalling mechanisms in heterogeneous cell populations, information theory is gaining more importance^19,20^. In contrast to mechanistic systems biology approaches^21–24^, information theoretic approaches enable analysing cellular signalling without complete knowledge of the nonlinear and complex structure of the underlying pathways. In information theory, transmission of a signal from a sender to a receiver via a noisy channel is analysed^25^. Application of information theoretic approaches to signalling pathways has primarily been used to determine Channel Capacities (CC)^26–30^. Here the signalling pathways are interpreted as channel and activation of transcription factors or downstream cellular responses are viewed as receiver. Channel Capacity represents the maximal number of different inputs (e.g. different cytokine concentrations) that can be discriminated by the receiver. The maximum number of inputs is calculated as 2 to the power of Channel Capacity_._

In addition, information theory also allows quantifying the mutual dependency of any two signalling events by calculating the Mutual Information (MI) of these two events. In contrast to other measures of dependency that are restricted to analysing linear dependencies such as correlation analysis, calculation of Mutual Information allows dealing with non-linear effects that are inherent to biological processes. That is why we use Mutual Information as a measure of correlation and in a second step as a measure for robustness. Robust signalling events depend on the presence of a stimulus but are independent of variation in expression of signalling proteins. In detail we calculate Mutual Information to determine whether and how IL-6-induced STAT3 activation is robust to variations in STAT3 protein copy number. High Mutual Information indicates that STAT3 phosphorylation is strongly influenced by variations in STAT3 expression, whereas low Mutual Information indicates that STAT3 phosphorylation is robust against variability in STAT3 expression.

In summary, we use information theoretic approaches to define mechanisms that facilitate robust STAT3 phosphorylation despite cell-to-cell variation in STAT3 expression and to determine how these mechanisms control Channel Capacity of IL-6-induced JAK/STAT signalling. We show that the feedback-inhibitor SOCS3 and phosphorylation of STAT3 at serine 727 enable robust activation of STAT3 by limiting STAT3 tyrosine phosphorylation. Additionally, robustness is ensured in the presence of low amount of cytokine and high extent of STAT3 expression. Of note, these mechanisms complement each other at different time scales and cytokine doses.

Channel Capacity of JAK/STAT signalling is reduced by cell-to-cell variability of STAT3 protein copy number, by low STAT3 expression and by mechanisms reducing STAT3 activation such as negative feedback and serine phosphorylation.

## Results

### IL-6 signalling results in heterogeneous STAT3 activation

**Fig. 1:**
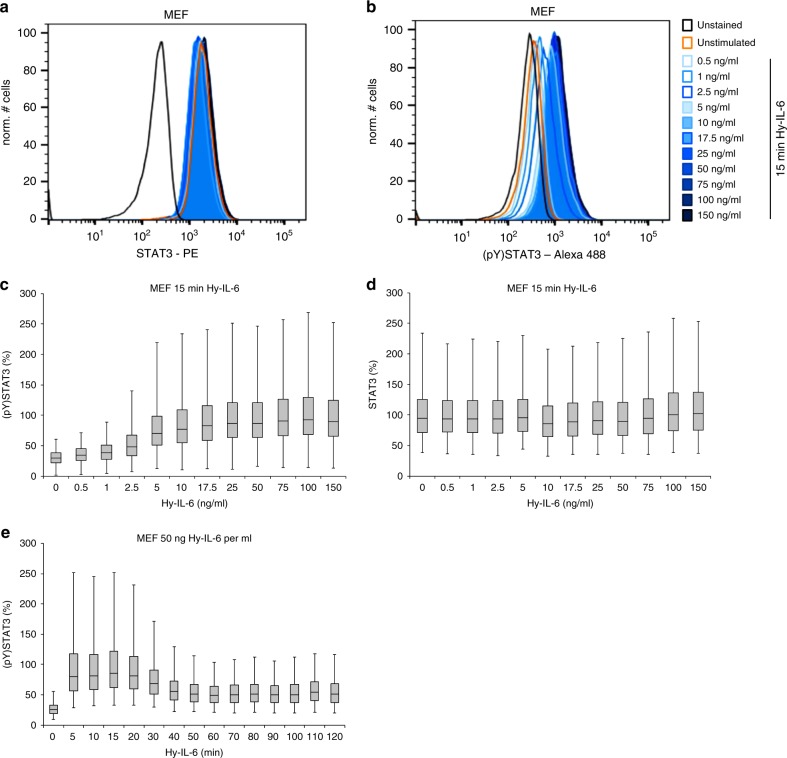
IL-6 signalling results in a rapid and dose-dependent heterogeneous STAT3 activation. **a**, **b** MEF cells were stimulated with increasing amount of Hy-IL-6 for 15 min. STAT3 expression and phosphorylation were evaluated by intracellular multiplex flow cytometry using specific fluorescent antibodies against STAT3 (**a**) and STAT3 (pY)705 (**b**). The flow cytometry gating strategy is shown in Supplementary Figure 1. Representative histograms of n = 3 independent experiments are shown. **c**, **d** MEF cells were stimulated with increasing amount of Hy-IL-6 for 15 min. STAT3 phosphorylation and expression were evaluated by intracellular multiplex flow cytometry using specific fluorescent antibodies against STAT3 (pY)705 (**c**) and STAT3 (**d**). For independent experiments, mean fluorescence of cells per cytokine dose was calculated. Maximal mean fluorescence in each experiment was normalised to 100 %. Data are pooled from n = 3 experiments. **e** MEF cells were stimulated with 50 ng Hy-IL-6 per ml for the indicated times. STAT3 phosphorylation was evaluated by intracellular flow cytometry using specific fluorescent antibodies against STAT3 (p)Y705. For independent experiments, mean fluorescence of cells per time point was calculated and maximal mean fluorescence in each experiment was normalized to 100 %. The data are from *n* = 3 experiments

IL-6-induced JAK/STAT signalling has extensively been analysed in cell populations^4^. To investigate the influence of cell-to-cell variability on JAK/STAT signalling, we developed a flow cytometric assay that allows for simultaneously analysing STAT3 expression and STAT3 tyrosine phosphorylation in single cells. Immortalised murine embryonal fibroblasts (MEF) were stimulated with increasing amounts of Hy-IL-6 for 15 min. MEF cells, like most other cells, do not express transmembrane IL-6 receptor. Thus, IL-6-induced STAT3 activation in MEF cells depends on trans-signalling. IL-6 and soluble IL-6 receptor form non-covalent complexes whose concentration is not trivially predictable^31^. In clear contrast the concentration of the fusion protein Hy-IL-6, used here, is known precisely, which is a prerequisite for dose-dependent studies. After stimulation, cells were fixed and stained with differentially labelled antibodies against STAT3 and Y705-phosphorylated STAT3. Single cell flow cytometry analyses of intracellular STAT3 reveal substantial differences in STAT3 expression within the cell-population, indicating that individual cells differ strongly with respect to STAT3 protein copy number. Heterogeneity of STAT3 expression is independent of stimulation with Hy-IL-6 (Fig. 1a, Supplementary Figure 1). Cell-to-cell heterogeneity of STAT3 protein expression was confirmed by immunofluorescence (Supplementary Figure 2a, b). To test whether STAT3 heterogeneity is influenced by immunostaining artefacts, we analysed expression of STAT3 in STAT3-deficient MEF cells stably transduced with STAT3-CFP by flow cytometry (Supplementary Figure 2c). The distribution of STAT3-CFP resembles the distribution of STAT3 expression as shown by immunostaining (Fig. 1a and Supplementary Figure 2a, b). Cell-to-cell heterogeneity of STAT3 expression is affected by extrinsic and intrinsic mechanisms including cell-cycle-phase^32^. We confirmed the increase of STAT3 protein expression during cell-cycle progression by detecting STAT3 expression and DNA content in parallel using flow cytometry (Supplementary Figure 3). Next, we asked whether cell-to-cell differences in STAT3 expression influence activation of STAT3.

Stimulation with Hy-IL-6 results in dose-dependent phosphorylation of STAT3 (Fig. 1b). As seen for STAT3 expression, the extent of STAT3 phosphorylation varies between individual cells. To compare our results with population-based analyses we pooled single cell data from 10,000 cells per condition. STAT3 phosphorylation increases dose-dependently in response to up to 25 ng Hy-IL-6 per ml (Fig. 1c) whereas STAT3 expression is not affected by stimulation (Fig. 1d). Additionally, we analysed the kinetics of Hy-IL-6-induced STAT3 phosphorylation for up to 120 min. IL-6 induces rapid phosphorylation of STAT3 that decreases in amplitude after 30 min (Fig. 1e). These pooled data are in line with analyses of IL-6-induced signalling with population-based methods^33^.

In summary, expression of STAT3 and IL-6-induced activation of STAT3 vary strongly between isogenic immortalised MEF cells, suggesting that cell-to-cell heterogeneity in STAT3 expression might influence signal transmission. This raises the question to what extent the amount of STAT3 in an individual cell affects the strength of cytokine-induced STAT3 phosphorylation in this cell.

### STAT3-Y705 phosphorylation is robust for weak stimuli

**Fig. 2:**
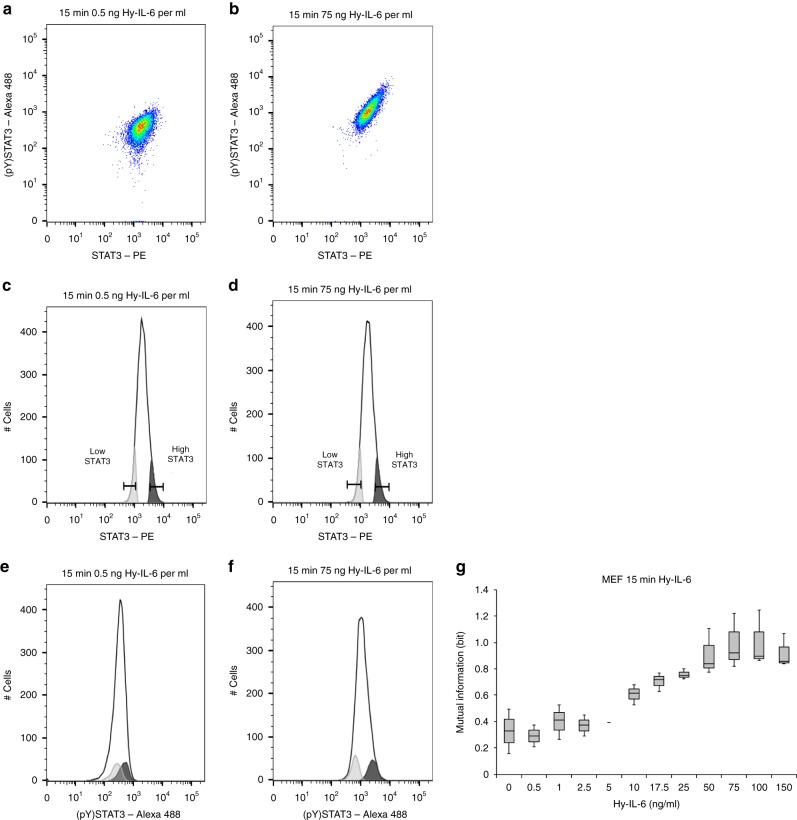
Robustness of STAT3 phosphorylation decreases with the strength of stimulation. **a**, **b** Pseudocolour plots depicting STAT3-Y705 phosphorylation and STAT3 expression in MEF cells stimulated with 0.5 ng Hy-IL-6 per ml for 15 min (**a**) or stimulated with 75 ng Hy-IL-6 per ml for 15 min (**b**). **c**, **d** Gating strategy to visualise dependency of STAT3 phosphorylation on STAT3 expression: Two sub populations including 10 % of MEF cells with the lowest (light grey) and the highest (dark grey) STAT3 expression, respectively, for cells stimulated with low amount (**c**) and high amount of cytokine (**d**). **e**, **f** STAT3 phosphorylation in the low STAT3 and high STAT3 expressing subpopulations was compared to overall STAT3 phosphorylation in the complete population (open histogram) in cells stimulated with low amount (**e**) and high amount of cytokine (**f**). Representative results of *n* = 3 independent experiments are shown. **g** Based on the data presented in Fig. 1c, d, Mutual Information between STAT3 expression and STAT3-Y705 phosphorylation in MEF cells stimulated for 15 min with Hy-IL-6 was calculated. The data are from *n* = 3 experiments

Our multiplexed analysis enables us to correlate STAT3 expression and STAT3 activation in single cells. Scatter plot analyses depicting STAT3 expression and STAT3 activation in cells stimulated with a low dose of Hy-IL-6 hint to a weak positive correlation of both parameters (Fig. 2a). However, STAT3 expression more obviously correlates with STAT3 activation in cells stimulated with a high dose of Hy-IL-6 (Fig. 2b). For detailed graphical analyses, we gated 10% of cells with lowest and highest STAT3 expression, stimulated with low and high amount of IL-6 (Fig. 2c, d) and determined STAT3 activation in these subpopulations (Fig. 2e, f). In cells stimulated with low amount of Hy-IL-6, the distributions of phosphorylated STAT3 in cells expressing high and low STAT3 numbers strongly overlap (Fig. 2e). However, in cells stimulated with high amount of Hy-IL-6, these distributions of STAT3 phosphorylation are clearly separated (Fig. 2f). Our observations suggest that, depending on the intensity of the stimulus, the amount of STAT3 in an individual cell influences the strength of STAT3 activation. IL-6-induced phosphorylation of STAT3 is a non-linear process^21^, and thus the calculation of a linear correlation between STAT3 expression and STAT3 activation does not yield insightful results. To circumvent this problem, we use Mutual Information to quantify the dependency of STAT3 activation on STAT3 protein expression. We calculated Mutual Information between STAT3 expression and STAT3 phosphorylation for 12 different doses of Hy-IL-6. As demonstrated in Fig. 2g, Mutual Information is low at low doses of cytokine and increases with increasing amount of cytokine until it reaches a plateau in response to 75 ng Hy-IL-6 per ml.

This indicates that for low to intermediate Hy-IL-6 concentrations the extent of STAT3 phosphorylation is robust against variability in STAT3 expression, whereas for high cytokine concentrations - that cause a strong phosphorylation of STAT3 - robustness is reduced.

In contrast to MEF cells, which do not express transmembrane IL-6 receptor and thus depend on trans-signalling, human hepatoma (HepG2) cells express IL-6 receptor and are responsive to classic signalling. We next tested whether robustness of IL-6 classic signalling-induced STAT3 phosphorylation against cell-to-cell variation in STAT3 protein copy number is also dose-dependent. We stimulated HepG2 cells with increasing equimolar doses of either IL-6 to induce classic signalling or Hy-IL-6 to induce trans-signalling. Both stimuli induce a dose-dependent phosphorylation of STAT3 (Supplementary Figure 4a), while STAT3 expression is not influenced by stimulation (Supplementary Figure 4b). As shown for MEF cells (Fig. 2g), Mutual Information between STAT3 expression and activation in HepG2 cells is low for low doses of IL-6 or Hy-IL-6 and increases with increasing amounts of cytokine, indicating reduced robustness at high concentration of cytokine (Supplementary Figure 4c).

In summary, both classic and trans-signalling-induced STAT3 activation is robust against differences in STAT3 expression for weak stimuli, whereas robustness is reduced for strong stimuli.

### Robustness of STAT3 activation depends on STAT3 expression

**Fig. 3:**
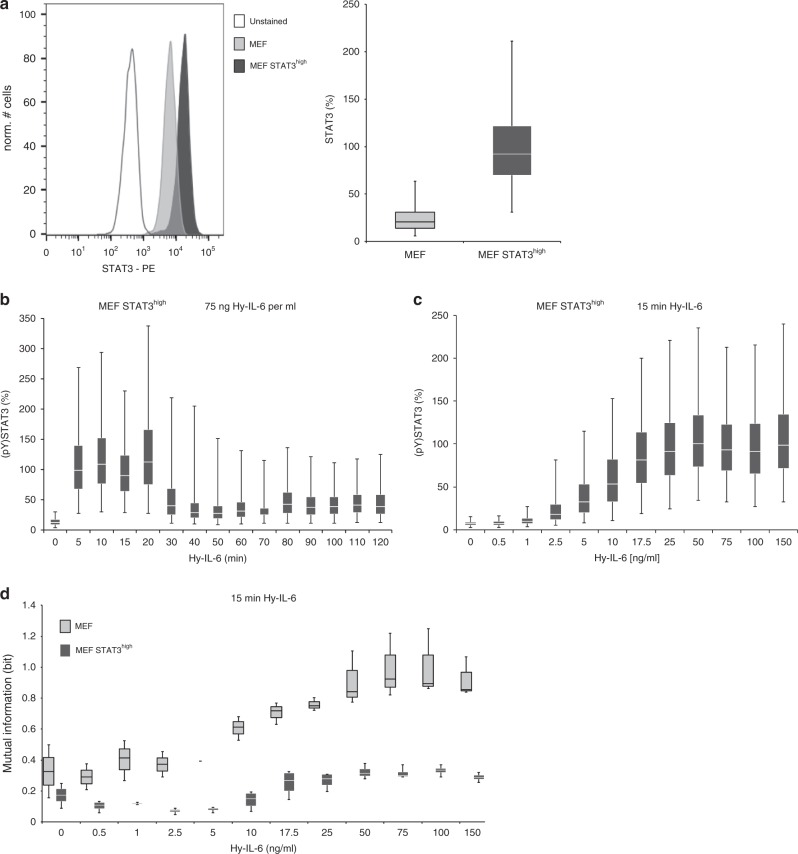
Robustness of IL-6-induced STAT phosphorylation increases with STAT3 expression. **a** MEF cells were stably transduced with cDNA for murine STAT3 to gain MEF STAT3^high^ cells. Expression of STAT3 in MEF and MEF STAT3^high^ cells was analysed by intracellular flow cytometry. A representative histogram is shown. For independent experiments mean fluorescence of cells was calculated. Maximal mean fluorescence in each experiment was normalised to 100 %. Box-and-whisker-plot depicts data from n = 3 experiments. **b** MEF STAT3^high^ cells were stimulated with 75 ng Hy-IL-6 per ml for the indicated times. STAT3 phosphorylation was evaluated by intracellular flow cytometry using specific fluorescent antibodies against STAT3 (pY)705. For independent experiments mean fluorescence of cells per cytokine dose was calculated. Maximal mean fluorescence in each experiment was normalised to 100 %. Data are pooled from n = 3 experiments. **c** MEF STAT3^high^ cells were stimulated with increasing amount of Hy-IL-6 for 15 min. STAT3 phosphorylation and expression were evaluated by intracellular multiplexed flow cytometry using specific fluorescent antibodies against STAT3 (pY)705 and STAT3 (Supplementary Figure 5). For independent experiments mean fluorescence of cells per cytokine dose was calculated. Maximal mean fluorescence in each experiment was normalised to 100 %. Data are pooled from n = 4 experiments. **d** Based on the data presented in Fig. 3c and Supplementary Figure 5, Mutual Information between STAT3 expression and IL-6-induced STAT3 phosphorylation in MEF STAT3^high^ cells stimulated for 15 min with Hy-IL-6 was calculated. The data are from *n* = 4 independent experiments. Overlay with Fig. 2g (MEF) for direct comparison of MEF cells and MEF STAT3^high^ cells

We observed that robustness against STAT3 protein copy number variation is reduced when STAT3 phosphorylation is high (Fig. 2g, Supplementary Figure 4c). We hypothesised that in conditions provoking strong STAT3 activation, the amount of available STAT3 restricts robustness of cytokine-induced STAT3 activation. To test this hypothesis, we increased STAT3 expression in MEF cells by stable overexpression of STAT3 (MEF STAT3^high^ cells). Single cell analyses demonstrated an average 3-fold overexpression of STAT3 in MEF STAT3^high^ cells (Fig. 3a) which is not affected by stimulation with Hy-IL-6 (Supplementary Figure 5). Furthermore, overexpression of STAT3 does not influence surface-expression of glycoprotein 130 (Supplementary Figure 6).

Stimulation of MEF STAT3^high^ cells with Hy-IL-6 induces a strong activation of STAT3 that decreases in amplitude after 30 min (Fig. 3b). Hy-IL-6-induced STAT3 phosphorylation in MEF STAT3^high^ cells increases dose-dependently in response to up to 25 ng Hy-IL-6 per ml (Fig. 3c) as already shown for MEF cells (Fig. 1b, c).

To investigate the influence of STAT3 overexpression on robustness of STAT3 activation we calculated the Mutual Information between STAT3 expression and STAT3 activation in MEF STAT3^high^ cells (Fig. 3d, dark grey boxes). In comparison to MEF cells (Fig. 2g and for direct comparison Fig. 3d, light grey boxes) Mutual Information in MEF STAT3^high^ cells stimulated with Hy-IL-6 for 15 min is strongly reduced and less dose-dependent. This demonstrates that increasing the amount of STAT3 increases robustness of STAT3 activation in cells stimulated with high cytokine concentrations.

In summary, the protein copy number of STAT3 is a limiting factor for robust STAT3 activation especially in the presence of high amount of cytokine.

### Robustness of late STAT3 phosphorylation depends on SOCS3

We observed robustness of early STAT3 phosphorylation in MEF cells stimulated with low to intermediate concentrations of cytokine, resulting in relatively weak STAT3 phosphorylation (Fig. 2g). On a time scale, STAT3 phosphorylation is limited by expression of the feedback-inhibitor SOCS3. Therefore, we hypothesised that reduction of STAT3 phosphorylation by SOCS3 might promote robustness of STAT3 activation at late time points. To test this hypothesis, we analysed robustness of STAT3 activation in SOCS3-deficient MEF cells (MEF SOCS3^-/-^).

**Fig. 4:**
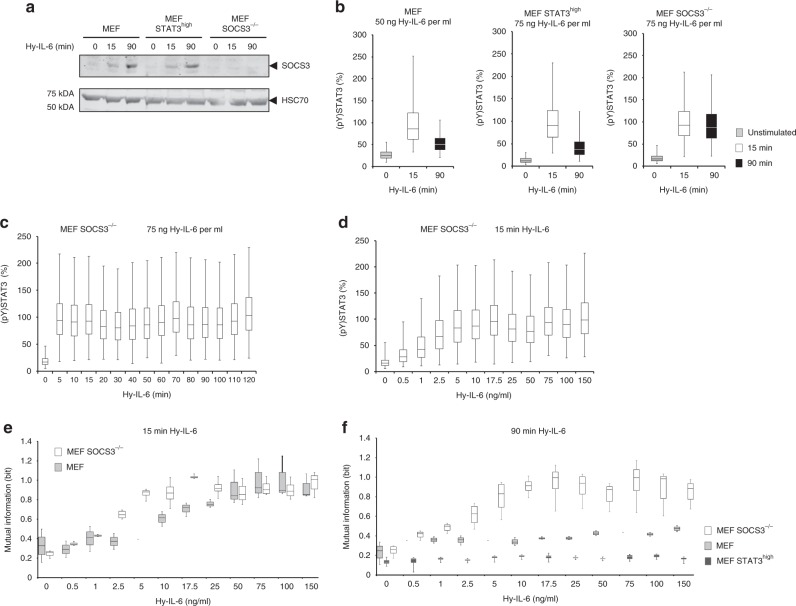
The SOCS3-negative feedback loop increases robustness of STAT3 activation against varying STAT3 expression. **a** MEF, MEF STAT3^high^, and MEF SOCS3^-/-^ cells were stimulated with 75 ng Hy-IL-6 per ml for the indicated times. SOCS3, and HSC70 protein expression were evaluated by Western blotting. A representative result of *n* = 6 independent experiments is shown. Uncropped Western blots are depicted in Supplementary Figure 7a, b. **b** MEF, MEF STAT3^high^, and MEF SOCS3^-/-^ cells were stimulated with 50 ng Hy-IL-6 per ml or with 75 ng Hy-IL-6 per ml for 15 and 90 min or left untreated. STAT3 phosphorylation was evaluated by intracellular flow cytometry. For independent experiments mean fluorescence of cells per time point was calculated. Maximal mean fluorescence in each experiment was normalised to 100 %. Data from *n* = 3 experiments. **c** MEF SOCS3^-/-^ cells were stimulated with 75 ng Hy-IL-6 per ml for the indicated times. STAT3 phosphorylation was evaluated by intracellular flow cytometry. **d** MEF SOCS3^-/-^ cells were stimulated with increasing amount of Hy-IL-6 for 15 min. STAT3 phosphorylation and expression were evaluated by intracellular multiplex flow cytometry using specific fluorescent antibodies against STAT3 (pY)705 and STAT3 (Supplementary Figure 8b). For independent experiments, mean fluorescence of cells per cytokine dose was calculated. Maximal mean fluorescence in each experiment was normalised to 100%. The data are from *n* = 4 experiments. **e** Based on Fig. 4d and Supplementary Figure 8b, Mutual Information between STAT3 expression and STAT3 phosphorylation in MEF SOCS3^-/-^ cells stimulated for 15 min with Hy-IL-6 was calculated. The data are from *n* = 4 independent experiments. Overlay with Fig. 2g (MEF) for direct comparison of MEF cells and MEF SOCS3^-/-^ cells. **f** Based on the data presented in Supplementary Figure 9 (for MEF), S10 (for MEF SOCS3^-/-^), and S11 (for MEF STAT3^high^), Mutual Information between STAT3 expression and IL-6-induced STAT3 phosphorylation in MEF (light grey), MEF SOCS3^-/-^ (white) and MEF STAT3^high^ (dark grey) cells stimulated for 90 min with Hy-IL-6 was calculated. The data are from *n* = 3, 4, and 4 independent experiments, respectively

In MEF and MEF STAT3^high^ cells, Hy-IL-6 induces SOCS3 expression within 90 min post stimulation. As expected, Hy-IL-6 does not induce SOCS3 expression in MEF SOCS3^-/-^ cells (Fig. 4a, Supplementary Figure 7). Consequently, STAT3 phosphorylation is reduced when SOCS3 is expressed in MEF and MEF STAT3^high^ cells. In contrast, STAT3 phosphorylation is not reduced in MEF SOCS3^-/-^ cells at 90 min of stimulation (Fig. 4b). In line with this, the detailed analyses of the kinetics of Hy-IL-6-induced STAT3 activation in SOCS3-deficient cells illustrates sustained STAT3 tyrosine phosphorylation (Fig. 4c). Stimulation of MEF SOCS3^-/-^ cells with Hy-IL-6 for 15 min reveals a dose-dependent STAT3 phosphorylation (Fig. 4d). STAT3 expression in MEF SOCS3^-/-^ cells and in MEF cells is comparable (Supplementary Figure 8a) and does not change in response to Hy-IL-6 (Supplementary Figure 8b). Also, surface-expression of glycoprotein 130 is not influenced by SOCS3 deficiency (Supplementary Figure 6).

We first calculated Mutual Information as a measure for robustness of STAT3 activation in MEF SOCS3^-/-^ cells stimulated for 15 min with Hy-IL-6 (Fig. 4e, white boxes). As SOCS3 is hardly expressed within the first 15 min of signalling, the results almost resemble those of MEF cells (Fig. 2g and for direct comparison Fig. 4e, light grey boxes) with STAT3 activation being robust against varying STAT3 expression for low to intermediate concentrations of cytokine. The slightly reduced robustness of STAT3 activation (Fig. 4e) in MEF SOCS3^-/-^ cells might be due to the weak expression of SOCS3 15 min post stimulation in MEF cells (Fig. 4a) or by increased basal levels of STAT3 phosphorylation in SOCS3-deficient MEF cells compared to MEF cells^34^.

To elaborate the impact of SOCS3 on the robustness of STAT3 activation, we analysed robustness of STAT3 activation 90 min post stimulation. At this time SOCS3 is strongly expressed (Fig. 4a) and the negative feedback efficiently limits STAT3 activation (Fig. 4b). We determined dose-dependent STAT3 activation and STAT3 expression in MEF (Supplementary Figures 9a, b) and MEF SOCS3^-/-^ cells (Supplementary Figure 10a, b) stimulated with Hy-IL-6 for 90 min. We calculated Mutual Information between STAT3 expression and activation for both cell lines. Mutual Information is strongly increased in MEF SOCS3^-/-^ cells especially in cells stimulated with high doses of Hy-IL-6 (Fig. 4f, white boxes) compared to MEF cells (Fig. 4f, light grey boxes). From this we conclude that reduced STAT3 phosphorylation caused by expression of the feedback-inhibitor SOCS3 results in enhanced robustness of STAT3 activation at late time-points.

To test whether overexpression of STAT3 also contributes to robustness of late STAT3 activation, we analysed the Mutual Information between STAT3 activation and STAT3 expression in MEF STAT3^high^ cells stimulated with Hy-IL-6 for 90 min (Supplementary Figures 11a, b). Indeed, overexpression of STAT3 not only increases the robustness of early STAT3 activation but also of late STAT3 activation in comparison to MEF cells (Fig. 4f).

In summary, both high STAT3 expression and low STAT3 activation, which is achieved by low doses of cytokine and by the SOCS3 feedback, increase robustness of late cytokine-induced STAT3 activation against variation in its expression.

### Serine 727 increases robustness of STAT3 activation

**Fig. 5:**
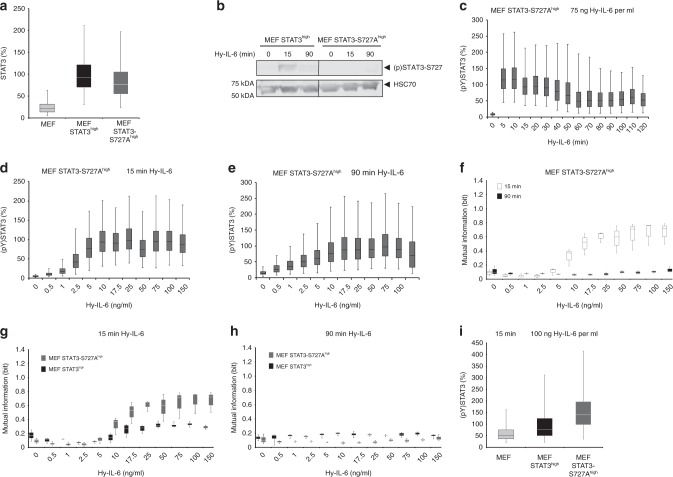
STAT3-S727 phosphorylation increases robustness of STAT3 phosphorylation against varying STAT expression. **a** STAT3 expression in MEF, MEF STAT3^high^, and MEF STAT3-S727A^high^ cells was evaluated by flow cytometry using fluorescent antibodies against STAT3. For independent experiments mean fluorescence of cells per cell line was calculated. Mean fluorescence of STAT3 expression in MEF STAT3^high^ cells was set to 100%. The data are from *n* = 3 experiments. **b** MEF STAT3^high^ and MEF STAT3-S727A^high^ cells were stimulated with 75 ng Hy-IL-6 per ml for the indicated times. STAT3-S727 phosphorylation and HSC70 expression were evaluated by Western blotting. A representative result of *n* = 6 independent experiments is shown. Uncropped Western Blots are shown in Supplementary Figure 12a, b. **c** MEF STAT3-S727A^high^ cells were stimulated with 75 ng Hy-IL-6 per ml for the indicated times. STAT3 phosphorylation was evaluated by flow cytometry. **d** MEF STAT3-S727A^high^ cells were stimulated with increasing amount of Hy-IL-6 for 15 min. STAT3 phosphorylation and expression were evaluated by flow cytometry using fluorescent antibodies against STAT3 (pY)705 and STAT3 (Supplementary Figure 13). The data are pooled from *n* = 3 experiments. **e** MEF STAT3-S727A^high^ cells were stimulated with increasing amount of Hy-IL-6 for 90 min and analysed as in d) (Supplementary Figure 14). For independent experiments, mean fluorescence of cells per cytokine dose was calculated. Maximal mean fluorescence in each experiment was normalised to 100 %. Data are from n = 3 experiments. **f** Based on data presented in Fig. 5d and Supplementary Figure 13, or Fig. 5e and Supplementary Figure 14, Mutual Information between STAT3 expression and IL-6-induced STAT3 phosphorylation in MEF STAT3-S727A^high^ cells stimulated with Hy-IL-6 for 15 min (white) or 90 min (black) was calculated. Data are from n = 3 independent experiments. **g** Overlay of Fig. 3d and Fig. 3f. **h** Overlay of Fig. 4f and Fig. 5f. **i** MEF, MEF STAT3^high^, and MEF STAT3-S727A^high^ cells were stimulated with 100 ng Hy-IL-6 per ml for 15 min. STAT3 phosphorylation in MEF, MEF STAT3^high^, and MEF STAT3-S727A^high^ cells was evaluated by flow cytometry. For independent experiments mean fluorescence of cells per cell line was calculated. Mean fluorescence of STAT3 phosphorylation in MEF STAT3^high^ cells was set to 100%. The data are from *n* = 3 experiments

IL-6-induced STAT3 serine 727 phosphorylation is discussed to be a fine tuner of IL-6-induced JAK/STAT signalling. That is why we hypothesised that cytokine-induced phosphorylation of S727 might contribute to robustness of STAT3 tyrosine phosphorylation. Specific pharmacological inhibition of IL-6-induced serine phosphorylation is troublesome as several kinases are discussed to be involved in S727 phosphorylation^15^. Therefore, we analysed STAT3 tyrosine phosphorylation in STAT3-deficient MEF cells stably reconstituted with STAT3-S727A (MEF STAT3-S727^high^ cells) and MEF STAT3^high^ cells. The mean STAT3 expression in MEF STAT3 S727A^high^ cells and in MEF STAT3^high^ cells is comparable (Fig. 5a) which allows us to compare robustness in these two cell lines independent of STAT3 expression level. Overexpression of STAT3-S727A furthermore does not influence surface-expression of glycoprotein 130 (Supplementary Figure 6). As expected, Hy-IL-6 does not induce S727 phosphorylation in MEF STAT3-S727A^high^ cells, whereas cytokine-induced phosphorylation of S727 is evident in MEF STAT3^high^ cells (Fig. 5b, Supplementary Figure 12a, b). The kinetics of Hy-IL-6-induced STAT3 tyrosine phosphorylation in MEF STAT3-S727A^high^ cells is rapid with maximal phosphorylation within 5 min and a decrease in amplitude after 15 min (Fig. 5c).

To calculate Mutual Information, we monitored dose-dependent STAT3 phosphorylation and expression in MEF STAT3-S727A^high^ cells stimulated for 15 min (Fig. 5d, Supplementary Figure 13) and 90 min (Fig. 5e, Supplementary Figure 14). As shown for MEF cells and MEF STAT3^high^ cells, STAT3 activation in MEF STAT3-S727A^high^ cells is more robust when stimulated with high doses of cytokine for 90 min than for 15 min (Fig. 5f), which is probably due to the active SOCS3-negative feedback.

In comparison to MEF STAT3^high^ cells (Fig. 3d and for direct comparison Fig. 5g, dark grey boxes) robustness of early STAT3 phosphorylation is clearly reduced in MEF STAT3-S727A^high^ cells (Fig. 5g, grey boxes). This difference is not apparent at 90 min (Fig. 5h for direct comparison). From these data, we conclude that STAT3 serine phosphorylation increases robustness of early STAT3 tyrosine phosphorylation at high doses of cytokine.

As robustness of STAT3 activation is affected by the strength of STAT3 tyrosine phosphorylation, we wondered whether phosphorylation of STAT3 at Y705 is influenced by mutation of S727 to alanine. As demonstrated in Fig. 5i, STAT3 phosphorylation is strongly increased in MEF STAT3-S727A^high^ cells compared to MEF STAT3^high^ cells. This indicates that serine phosphorylation of STAT3 negatively affects STAT3 tyrosine phosphorylation. The increase in STAT3 tyrosine phosphorylation might be causative for the reduced robustness of STAT3 activation against varying STAT3 protein expression in MEF STAT3-S727A^high^ cells.

In summary, STAT3-S727 phosphorylation increases robustness of early STAT3 activation especially in the presence of high doses of cytokine, which is a so far unknown function of STAT3 serine phosphorylation.

### Variability in STAT3 expression reduces Channel Capacity

So far, our studies focussed on mechanisms enabling robustness of STAT3 activation against varying STAT3 expression in single cells. Next, we asked whether cell-to-cell variability affects the amount of information transmitted through the JAK/STAT pathway. To this end we calculated Channel Capacity, which is a measure for the maximal number of different input values, i.e. cytokine concentrations, that can be discriminated by the receiver, here defined as STAT3 tyrosine phosphorylation.

**Fig. 6:**
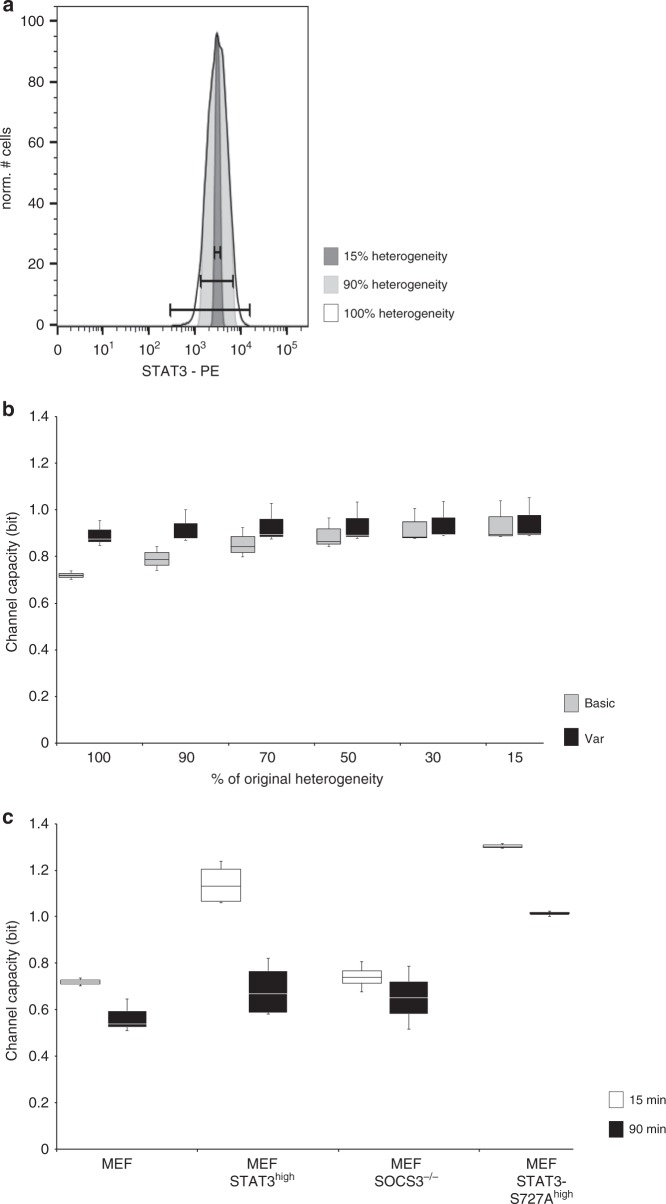
Channel Capacity of IL-6-induced JAK/STAT signalling. **a** Based on the data presented in Fig. 1 (MEF), the heterogeneity of STAT3 expression in the cell population is reduced by stepwisely excluding cells from the analysis. Starting with exclusion of cells that express the 5% highest and 5% lowest amount of STAT3 (90% residual variability) heterogeneity is reduced until only cells with mean STAT3 expression ± 7.5% were left (15 % residual variability). **b** Channel capacity of JAK/STAT signalling induced by Hy-IL-6 for 15 min in MEF cells was calculated. Channel Capacity basic and Channel Capacity var were calculated for cells with 100, 90, 70, 50, 30, and 15% residual variability of STAT3 expression. The data are from *n* = 3 independent experiments. **c** Based on the data presented in Fig. 1c (MEF), Fig. 3c (MEF STAT3^high^), Fig. 4d (MEF SOCS3^-/-^), and Fig. 5d (MEF STAT3-S727A^high^) Channel Capacity of JAK/STAT signalling induced by Hy-IL-6 for 15 min was calculated. Based on the data presented in Supplementary Figure 9a (MEF), Supplementary Figure 10a (MEF SOCS3^-/-^), Supplementary Figure 11a (MEF STAT3^high^), and Fig. 5e (MEF STAT3-S727A^high^), Channel Capacity of JAK/STAT signalling induced by Hy-IL-6 for 90 min was calculated. Data are from n = 3, 4, 4, and 3 independent experiments, respectively

Our single cell analyses revealed that STAT3 expression varies strongly between individual cells (Fig. 1a). We hypothesised that this variability of STAT3 expression influences Channel Capacity. To test this, we reduced the heterogeneity of STAT3 expression in a cell population by step wisely excluding cells from the analysis. We started with excluding cells that express the 5% highest and 5% lowest amount of STAT3 (90% residual heterogeneity) until only cells with mean STAT3 expression ± 7.5% (15% residual heterogeneity) were included in the analysis (Fig. 6a). Next, we calculated Channel Capacity for these subpopulations. Reducing the variability of STAT3 expression increases Channel Capacity (Channel Capacity basic) from 0.7 (100% heterogeneity) to 1 bit (15% heterogeneity) (Fig. 6b, light grey bars). This indicates that the heterogeneity of STAT3 expression limits the amount of information transmitted through the signalling pathway.

We hypothesised that this is caused by the lack of robustness of STAT3 activation against variation in STAT3 protein copy number (Fig. 2g). Consequently, cell-to-cell heterogeneity in STAT3 expression causes diversity in STAT3 activation and thus blurs dose-dependency of STAT3 phosphorylation. To test this, we re-calculated Channel Capacity for the different subpopulations with an algorithm that accounts for the dependency of STAT3-phosphorylation on STAT3 expression (Channel Capacity var). Indeed, including the information on STAT3 expression results in Channel Capacities independent of the heterogeneity of the cell population (Fig. 6b, dark grey bars). This supports our hypothesis that the sensitivity of STAT3 phosphorylation towards changes in STAT3 expression reduces Channel Capacity in heterogenic cell populations.

Next, we asked whether mechanisms defined by Mutual Information analyses to increase robustness of STAT3 activation will also affect capacity of JAK/STAT signalling. Channel Capacity of early Hy-IL-6-induced JAK/STAT signalling in MEF cells is 0.7 bit. When STAT3 expression is increased (MEF STAT3^high^ and MEF STAT3-S727A^high^ cells) Channel Capacity at 15 min of stimulation is strongly increased (Fig. 6c, white bars). This suggests that increasing robustness of early STAT3 activation by increasing STAT3 expression also facilitates higher Channel Capacity.

Knock out of SOCS3 (MEF SOCS3^-/-^) which does not influence STAT3 expression (Supplementary Figure 8) and early STAT3 activation (Fig. 4b), does not influence Channel Capacity early after stimulation (Fig. 6c, white bars). In contrast, SOCS3 expression reduces the extent of late STAT3 phosphorylation (Fig. 4b). In line with the reduced STAT3 phosphorylation at late time points, Channel Capacity at 90 min is reduced compared to Channel Capacity at 15 min in cell lines, where the SOCS3 feedback-loop is active (MEF, MEF STAT3^high^, and MEF STAT3-S727A^high^ cells). In MEF SOCS3^-/-^ cells Channel Capacity is however identical at 15 and 90 min post stimulation (Fig. 6c, dark bars). These results indicate that the negative feedback contributes to the timely orchestration of information transmitted through the JAK/STAT pathway.

To further test our hypothesis that mechanisms increasing tyrosine phosphorylation enhance Channel Capacity, we compared Channel Capacity in MEF STAT3^high^ cells and MEF STAT3-S727A^high^ cells (Fig. 5i). In MEF STAT3-S727A^high^ cells both early and late Channel Capacity are higher than in MEF STAT3^high^ cells (Fig. 6c). Thus, serine 727 phosphorylation in STAT3 decreases Channel Capacity, most probably by limiting tyrosine phosphorylation.

In summary, Channel Capacity of Hy-IL-6-induced JAK/STAT signalling is limited by cell-to-cell variability in STAT3 expression. Of note, Channel Capacity of JAK/STAT signalling is additionally affected by the same mechanisms that govern robustness of STAT3 activation. However, not all mechanisms affect Channel Capacity and robustness in the same direction. On one hand, an increase in STAT3 expression both increases Channel Capacity and robustness, on the other hand, an increase in STAT3 tyrosine phosphorylation by mutation of serine 727 to alanine reduces robustness but increases Channel Capacity. On a time scale, Channel Capacity of JAK/STAT signalling is reduced at late time points by negative feedback through SOCS3.

## Discussion

Heterogenous protein expression and post-translational modifications are common features of cell populations. Analysis of heterogeneous expression of cytokine receptors has revealed tremendous influence of cell-to-cell variability on cellular signalling^35^. In the present study, we show heterogeneous expression of the transcription factor STAT3 and IL-6-induced tyrosine phosphorylation of STAT3 by multiplexed flow cytometry. We use the information theoretic measure Mutual Information to determine mechanisms enabling robust activation of STAT3 by IL-6 classic and trans-signalling, despite significant cellular heterogeneity in STAT3 expression. To our knowledge, for the first time, we show that different mechanisms enabling robustness complement each other at different time scales and cytokine doses. Early Hy-IL-6-induced STAT3 activation is robust as long as cytokine concentrations are low to intermediate. Robustness at high cytokine concentrations is ensured by either increasing STAT3 expression or by limiting STAT3 phosphorylation. By decreasing tyrosine phosphorylation, STAT3 serine 727 phosphorylation contributes to robustness of early STAT3 activation, which is a so far unknown function of this regulatory post-translational modification. At late time-points of JAK/STAT signalling, the feedback-inhibitor SOCS3 increases robustness of STAT3 activation.

So far mechanisms contributing to negative regulation of JAK/STAT signalling such as SOCS3 expression, receptor internalisation, cytoplasmic and nuclear phosphatases, and transcriptional co-regulators such as protein inhibitors of activated STATs (PIAS) were primarily discussed to prevent overshooting signalling contributing to severe (auto-)inflammatory and proliferative diseases^7^. Our data now strongly supports the hypothesis that these mechanisms are also involved in sustaining robust signalling despite cell-to-cell heterogeneity. In the future, analysing the involvement of other regulatory mechanisms of STAT3 activation and STAT3 gene expression will identify further mechanisms ensuring robustness.

Our studies focussed on robustness of IL-6-induced STAT3 phosphorylation. IL-6 is a pleiotropic cytokine that, depending on the target cell, induces the expression of a plethora of target genes that can even have opposing functions^3,36,37^. So far it is unknown how cell-to-cell heterogeneity in STAT3 expression and activation contributes to these downstream events. However, the raising advent of single cell methods will allow us addressing mechanisms of robustness of cellular responses downstream of STAT3 activation in the future.

Identifying robust and sensitive components in signalling pathways enables definition of molecular targets for therapeutic intervention. Robustness against perturbations limits therapeutic interventions whereas sensitive reactions are promising therapeutic targets^38^. We show that robustness of STAT3 activation depends on the extent of STAT3 expression and the extent of STAT3 activation. Single cells characterized by high STAT3 expression or low STAT3 activation are robust against variability in STAT3 expression. Analyses of robust parameters in JAK/STAT signalling has so far been founded on laborious sensitivity analyses of complex mechanistic computational models^39,40^. Sensitivity analysis of IL-13-induced JAK/STAT5 signalling supports our findings by revealing STAT5 expression and phosphorylation among the highly sensitive parameters in JAK/STAT signalling^41^. Our Mutual Information approach therefore offers a straightforward method to determine robustness if multiplexed single cell data is available. The importance of STAT expression and phosphorylation for ensuring robustness in both IL-13-^41^ and IL-6-induced JAK/STAT signalling could point to the fact that mechanisms ensuring robustness of activation are conserved within the seven members of the family of STAT proteins and the plethora of JAK/STAT activating cytokines, interferons, and growth factors^42^.

The importance of STAT3 tyrosine phosphorylation for STAT3 dimerisation, nuclear translocation and transcriptional activity is well accepted. The physiological and pathophysiological consequences of STAT3 serine phosphorylation are however less explicit. Mice expressing STAT3-S727A display an unclear phenotype. Lethality is increased and growth decreased. The number of thymocytes is reduced but STAT3-dependent acute-phase response is not altered in these mice^43^. STAT3-S727 phosphorylation contributes to non-canonical functions of STAT3 in the mitochondria^9,44^, reduces STAT3 tyrosine phosphorylation^8^, DNA binding^12^, and transcriptional activity^12^. Reduced STAT3-Y705 phosphorylation might be explained by enhanced dephosphorylation of Y705 via phosphatases such as TC45^45^. Our data support this by showing that IL-6-induced STAT3 phosphorylation is increased in MEF cells expressing STAT3-S727A (Fig. 5i). Additionally, we show that STAT3 serine phosphorylation enables robustness of STAT3 tyrosine phosphorylation, which gives serine 727 phosphorylation a so far undescribed highly relevant physiological function.

In the second part of this paper, we determined Channel Capacity of JAK/STAT signalling. Channel Capacity of IL-6-induced JAK/STAT signalling is below 1 bit (Fig. 6). This indicates that analysing a single cell in a heterogeneous population merely allows discriminating whether this cell has seen low or high amount of cytokine. Interestingly, limited information transfer appears as a general feature of signalling pathways. Restricted information transfer has been shown for growth factor- and gonadotropin releasing hormone (GnRH)-induced mitogen-activated protein kinase signalling^27,29^, for transforming growth factor β (TGF-β)-dependent SMAD activation^26^,  and for Trail-induced apoptosis^28^. Of note, Channel Capacity of all analysed pathways might be higher if cells integrate additional information than considered in experimental settings. Several hypotheses have been elaborated to explain limited Channel Capacity in these pathways, and how reliable information transmission is possible despite low Channel Capacity. Information transmission can be increased by integration of several outputs^46^, by processing fold-changes rather than absolute changes^26^, by the existence of multiple gene-copies^47^, and by interpreting the dynamics of signalling^48^.

Here we show that information transmission through IL-6-induced JAK/STAT signalling is limited by cell-to-cell variability in STAT3 expression, low STAT3 protein expression, STAT3-S727 phosphorylation and on a time scale by the feedback-inhibitor SOCS3. Transmission of information within this pathway can be increased by enhancing STAT3 expression or activation (Fig. 6c). SOCS3-negative feedback restricts Channel Capacity of late IL-6-induced JAK/STAT signalling (Fig. 6c). The influence of negative feedback on Channel Capacity depends on the biological context. In gonadotropin releasing hormone-induced mitogen-activated protein kinase signalling information transfer is maximal at intermediate feedback strength^27^. In epidermal growth factor-induced mitogen-activated protein kinase signalling a negative feedback even increases Channel Capacity by suppressing basal network activity^30^. We show that increasing STAT3 protein expression enhances Channel Capacity of JAK/STAT signalling (Fig. 6c). The observation that increased numbers of signalling proteins enhance Channel Capacity has also been made in other systems. Having two copies of a gene coding for a signalling protein, under certain conditions increases the expression of the signalling protein and thereby information transmission^47^.

Interestingly, all mechanisms defined here to increase Channel Capacity (i.e. lack of STAT3-S727 phosphorylation, lack of SOCS3-mediated feedback, and overexpression of STAT3) are involved in the development of severe proliferative disorders. Expression of STAT3-S727A in glioma increases survival, proliferation and invasion^49^. Reduced SOCS3 expression, caused e.g. by hypermethylation of the SOCS3 promoter^50^ and overexpression of STAT3, caused e.g. by amplification of the STAT3 gene or by epigenetic modifications, are frequently found in solid cancers^51^. Increased information transmission might therefore result in molecular misconception that contributes to carcinogenesis. In line with this hypothesis in multi-cellular organisms a trade-off between information transfer in single cells and cell-populations exists. Low information transfer on the single cell level is a prerequisite for high information transfer on the population level^28^. Increasing Channel Capacity on the single cell level might disturb this trade-off and thereby contribute to disease development.

## Methods

### Cell Culture

Human hepatoma (HepG2) cells were directly purchased from DSMZ, Germany, No: ACC180. Murine embryonal fibroblasts (MEF) STAT3^fl/fl^ and MEF STAT3^-/-^ cells were a generous gift from Prof. V. Poli (University of Turin, Italy). MEF STAT3^high^, MEF STAT3-S727A^high^ cells were generated within the scope of this paper. MEF SOCS3^-/-^ cells were a generous gift from Prof. A. Yoshimura (Keio University Tokyo, Japan). MEF STAT3-CFP cells were a generous gift from Prof. G. Müller-Newen (RWTH Aachen, Germany).

Immortalised MEF, MEF STAT3^-/-^, and MEF SOCS3^-/-^ cell lines were grown in DMEM (Thermo Fisher Scientific, Waltham, MA, USA) supplemented with 10 % FCS (Thermo Fisher Scientific), streptomycin and penicillin (each 100 µg per ml solvent, Thermo Fisher Scientific) at 37 °C in a water saturated atmosphere containing 5 % CO_2_. MEF STAT3^fl/fl^ cells were retrovirally transduced with cDNA for murine STAT3 to generate MEF STAT3^high^ cells that stably overexpress STAT3. MEF STAT3^-/-^ cells were retrovirally transduced with cDNA for murine STAT3-S727A to generate MEF STAT3-S727A^high^ that stably overexpress STAT3-S727A. MEF STAT3^high^ and MEF STAT3-S727A^high^ cells were grown in DMEM supplemented with 10% FCS, 2 µg puromycin per ml (Carl Roth, Karlsruhe, Germany), streptomycin, and penicillin (each 100 µg per ml) at 37 °C in a water saturated atmosphere containing 5% CO_2_. All experiments were performed between passage numbers 5–20.

### Stimulation of cells

A total of 10^6^ cells were cultured on a 6 cm dish for 24 h. Prior to stimulation, cells were washed with PBS and subsequently starved in 2 ml medium without FCS and antibiotics for 2 h. Cells were treated with Hy-IL-6 (Conaris, Kiel, Germany) as indicated in the figures.

### Antibodies

Antibodies used for flow cytometry: Alexa Fluor 488 Mouse anti-STAT3 (pY705) (Clone 4/P-STAT3), BD Phosflow, Franklin Lakes, NJ, USA, Catalog No. 557814, Lot: 4003621; PE Mouse anti-STAT3 (Clone M59-50), BD Phosflow, Catalog No. 560391, Lot: 7046704; Mouse anti-gp130 (Clone BR-3), Hölzel Diagnostics, Cologne, Germany, Catalog No. 852.060.000, Lot:P11016D6.

Antibodies used for immunodetection of Western Blots: Rabbit anti-STAT3 (pY705) (Clone D3A7), Cell Signaling Technology, Cambridge, UK, Catalog No. 9145, Lot: 34; Rabbit anti-STAT3 (pS727) (Polyclonal), Cell Signaling Technology, Catalog No. 9134, Lot: 21; Mouse anti-STAT3 (Clone 124H6), Cell Signaling Technology, Catalog No. 9139, Lot: 10; Rabbit anti-SOCS3 (Clone C204), Immuno-Biological Laboratories, Fujioka, Japan, Catalog No. 18391, Lot: 0G-901; Mouse anti-HSC70 (Clone 1F2-H5), StressMarq Biocsciences, Victoria, Canada, Catalog No. SMC-151, Lot: 0706; Goat anti-Mouse DyLight 633 (polyclonal), Thermo Fisher Scientific, Catalog No. 815-968-0747, Lot: NB167398; Goat anti-Rabbit DyLight 550 (polyclonal), Thermo Fisher Scientific, Catalog No. 815-968-0747, Lot: NB165012.

### Western blotting

Cells were lysed in RIPA lysis buffer (50 mM Tris-HCl, pH 7.4, 150 mM NaCl, 0,5 % NP-40, 15 % Glycerol, supplemented with 10 µg of each aprotinin, leupeptin and pepstatin per ml as well as 0.8 µM Pefabloc (Roche, Mannheim, Germany), 1 mM NaF, and 1 mM Na_3_VO_4_). Protein concentration was determined using Bradford Assay according to manufacturer´s instructions (Carl Roth). Proteins were separated by SDS-Page before transfere to a nitrocellulose membrane (0.2 µm, GE Healthcare, Chicago, IL, USA). Antigens were detected by incubation with specific primary antibodies (1:1000) and subsequent incubation with infrared-fluorescent-dye (IRDye)-coupled secondary antibodies (1:10000 in TBSN) (LI-COR Biosciences, Lincoln, NE, USA). Detection was performed using Odyssey Infrared Imaging System (LI-COR). Western Blot data were recorded and analysed using Image Studio Lite (LI-COR), Version 5.2.

### Flow cytometry

All MEF cells were starved in DMEM without FCS and antibiotics for 2 h. After stimulation with Hy-IL-6, cells were detached from the cell culture dish with 1 ml Accutase (Biowest, Nuaillé, France Cat. No. L0950-100). For intracellular staining cells were fixed. Therefore, 100 µl of the cell suspension was mixed with 100 µl paraformaldehyde (4 %) and incubated at 37 °C for 10 min followed by centrifugation at 230 g, 4 °C for 5 min. Cell pellets were suspended in ice cold 90 % methanol and incubated on ice for 10 min. Subsequently, cells were washed twice with cold BSA-EDTA-Buffer (2 % BSA, 2 mM EDTA in PBS) and incubated with fluorophore-coupled antibodies (1:200) overnight. Cells were washed for two times in BSA-EDTA Buffer before FACS analysis. 10,000 individual cells were measured per condition. Flow Cytometry Data were recorded on a BD FACS Canto II equipped with 3 lasers (405 nm, 488 nm, 663 nm, Firmware Version 1.47) using FACS Diva (BD Biosciences), Version 6.1.3. Data were analysed using FlowJo (Treestar, Ashland, OR, USA), Version 10. FCS files were converted to Microsoft Excel files. For representation data from independent experiments as indicated in the figure legends were normalised to 100 % and pooled. Centre line of Box-and-whisker diagrams depicts median. Box limits are the 25 and 75 % quantiles. Whisker depict the 2.5 % and the 97,5 % quantiles.

### Mutual Information

Mutual Information (MI) is used to analyse how much information can be inferred about a random variable by measuring another random variable. Mutual Information is zero if the variables are unrelated, and positive if there is a dependency. It was computed by the formula:^46^

$${\mathrm{MI}}(S;R) = {\int\!\!\!\!\!\int} {p(S,R)\log _2\frac{{p(S,R)}}{{p(S)p(R)}}dRdS}$$

*R* and *S* denote the two random variables to be analysed, in our case the extent of STAT3 phosphorylation and STAT3 expression, respectively. *p(S,R)* is the joint probability density of these two variables and *p(S)* and *p(R)* are the marginal probability densities.

The computation was done with a custom-made Python script using the statistics and integration modules in the “scipy” package (version 0.15.1, Enthought, Austin, TX, USA). All probability densities were approximated by kernel density estimation^52^, and the integration was performed with the “quadpack” library.

### Channel Capacity

A closely related measure to Mutual Information is the Channel Capacity, which is defined as the maximal Mutual Information over all possible distributions of the signal (S)

$${\rm{CC}}(S;R) = \max _{{\mathrm{p}}\left( {\mathrm{S}} \right)}{\mathrm{MI}}\left( {S;R} \right),$$

where, as in the definition of mutual information, it is measured in bits and *S* and *R* are random variables. In our case, we measured Channel Capacity between simulation level of a single cytokine (S) and the amount of phosphorylated STAT3 (R). Firstly, continuing the interpretation of Mutual Information, it can be treated as the maximal (potential) information transfer between S and R. Secondly, the value 2^*CC*^ can be also interpreted as the number of states of S that can be distinguished with high confidence using knowledge of the values of R^25,53^.

Channel Capacity has been estimated in R (R version 3.3.0) using a package available at GitHub repository: https://github.com/sysbiosig/SLEMI, which is based on statistical learning methods and Monte Carlo for efficient computation.

### Data exclusions

Only experiments were technical errors occurred (i.e. unsuccessful stimulation or staining) were excluded from analysis. Data sets were Mutual Information was < 0 were excluded from analyses, as in theory Mutual Information should be higher or equal to 0. Most presumably these results were caused by numerically ill conditioned data sets.

### Code availability

Custom Code for calculation of Mutual Information can be accessed at: https://github.com/swaldherr/il6-mutual-information. Custom Code for calculation of Channel Capacity can be accessed at: https://github.com/sysbiosig/SLEMI.

## Supplementary information

Supplementary Information

## Data Availability

The data sets generated and analysed during the current study are available in: https://github.com/swaldherr/il6-mutual-information.
