# Supplementary material for: Robustness and Information Transfer within IL-6-induced JAK/STAT Signalling
Source: Commun Biol. 2019 Jan 18;2:27. doi: 10.1038/s42003-018-0259-4 (PMC6338669; doi:10.1038/s42003-018-0259-4)
Supplement: Supplementary file 1 — Supplementary Information [file 42003_2018_259_MOESM1_ESM.pdf]

S1

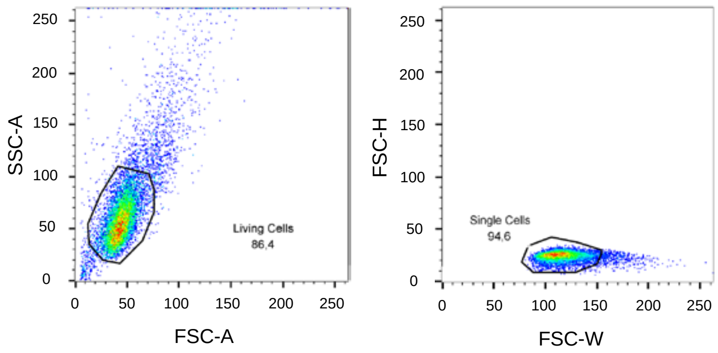

Supplementary Figure 1. Gating Strategy for selection of single MEF cells

Living cells were gated from a SSC-A against FSC-A plot. From this population single cells were gated by plotting FSC-H against FSC-W and gating on cells with low FSC-W. Only living single cells were subsequently analysed. Flow Cytometry Data were recorded on a BD FACS Canto II equipped with 3 lasers (405 nm, 488 nm, 663nm, Firmware Version 1.47) using FACS Diva (BD Biosciences), Version 6.1.3.Data were analysed using FlowJo (Treestar, Ashland, OR, USA), Version 10.

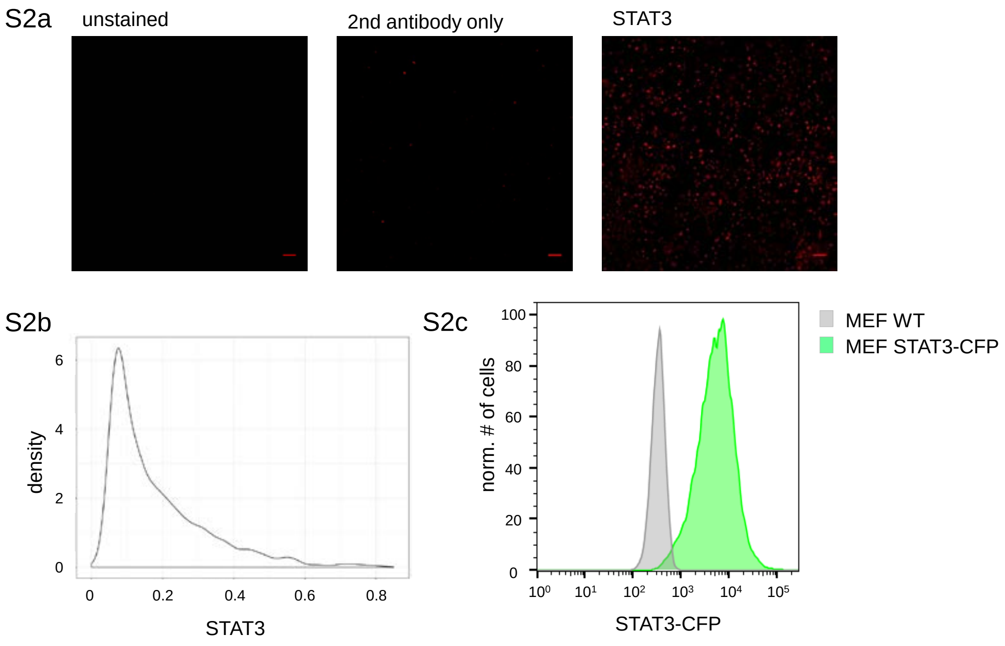

Supplementary Figure 2. Cell-to-cell heterogeneity in STAT3 protein expression

a) MEF cells were seeded on poly-L-lysine coated cover slides. 24 h later cells were washed 2x with PBS^{++} (1 mM $MgCl_2$, 0.1 mM $CaCl_2$) and subsequently fixed with 90 % methanol (15 min, -20 °C). Permeabilisation was achieved with PBS^{++T} (1 mM $MgCl_2$, 0.1 mM $CaCl_2$, 0.1 % Triton-X) for 5 min. Subsequently cells were quenched for 5 min with 0.05 M NH_4Cl and blocked for 1 h with 1 % BSA/PBS^{++T}. Cells were stained overnight in Anti-STAT3 antibody (Clone 124H6, Catalog No. 9139, Lot 10, Cell Signaling technologies, 1:200 in 0.2 % BSA/PBS^{++T}). After washing cells were stained for 1 h with Anti-Mouse-PE antibody (polyclonal, Catalog No. 115-116-146, Lot: 120701, Dianova, 1:200 in 0.2 % BSA/PBS++T). Control cells were either left unstained (unstained) or stained only with secondary antibody (2nd antibody only). Imaging was performed with a laser scanning microscope (63 x objective, LSM 700, Zeiss, Jena, Germany). PE was excited using laser light of 555 nm. Emission was detected in the range of 560 -700 nm with a pinhole size of 8.5 au and a PMT voltage gain of 600. Pictures were taken using Zen Software (Zeiss, version: 2010). The scale bar represents 100 µm. **b)** Cells were identified with an automated algorithm using CellProfiler (version: 3.1.5) [1]. Specifically, a global approach was used with a minimum cross entropy method for the choice of threshold (smoothing scale = 1.3488, correction factor = 1). Clumped objects were distinguished by their shape. Additionally, too big objects (diameter above 250 pixels) were discarded. For each cell, median fluorescence intensity of the cell's interior was used as a proxy of STAT3 concentration. Cells stained with only the secondary antibody were quantified by applying a similar strategy and used for background correction. The graph shows kernel density estimator of the distribution of STAT3 fluorescence intensity (after correction) obtained from 4594 single cells. **c)** MEF cells and STAT3-deficient MEF cells stably expressing STAT3-CFP were analysed by flow cytometry. Flow Cytometry Data were recorded on a BD FACS Canto II equipped with 3 lasers (405 nm, 488 nm, 663nm, Firmware Version 1.47) using FACS Diva (BD Biosciences), Version 6.1.3.Data were analysed using FlowJo (Treestar, Ashland, OR, USA), Version 10. Histogram shows a representative result of n = 2 experiments.

S3

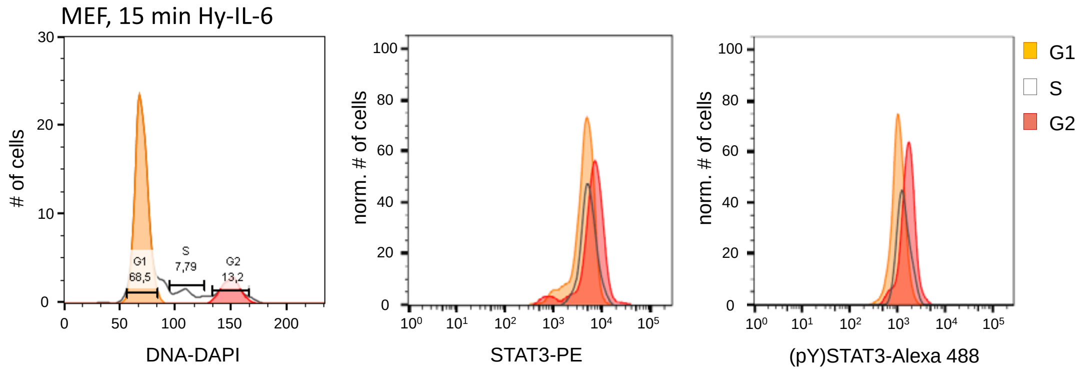

Supplementary Figure 3. STAT3 expression increases with cell-cycle phase

10^6 MEF cells were cultured on a 6 cm dish for 24 hours. After starvation for 2 h MEF cells were stimulated for 15 min with 25 ng Hy-IL-6 per ml. Subsequently, cells were detached from the cell-culture dish with 1 ml Accutase (Biowest, Nuaillé, France Cat. No. L0950-100). 100 µl of the cell-suspension was mixed with 100 µl paraformaldehyde (4 %) and incubated at 37 °C for 10 min followed by centrifugation at 230 g, 4 °C for 5 min. Cell pellets were suspended in ice-cold 90 % methanol and incubated on ice for 10 min. Subsequently, cells were washed twice with cold BSA-EDTA-Buffer (2 % BSA, 2 mM EDTA in PBS) and incubated with fluorophore-coupled antibodies (1:200) overnight. Antibodies used for flow cytometry: Alexa Fluor 488 Mouse anti-STAT3 (pY705) (Clone 4/P-STAT3), BD Phosflow, Franklin Lakes, NJ, USA, Catalog No. 557814, Lot: 4003621; PE Mouse anti-STAT3 (Clone M59-50), BD Phosflow, Catalog No. 560391, Lot: 7046704. Cells were washed again for two times in BSA-EDTA Buffer. Directly before analyses cells were incubated with DAPI (1: 10,000 in PBS-EDTA Buffer). Flow Cytometry Data were recorded on a BD FACS Canto II equipped with 3 lasers (405 nm, 488 nm, 663nm, Firmware Version 1.47) using FACS Diva (BD Biosciences), Version 6.1.3. Data were analysed using FlowJo (Treestar, Ashland, OR, USA), Version 10.

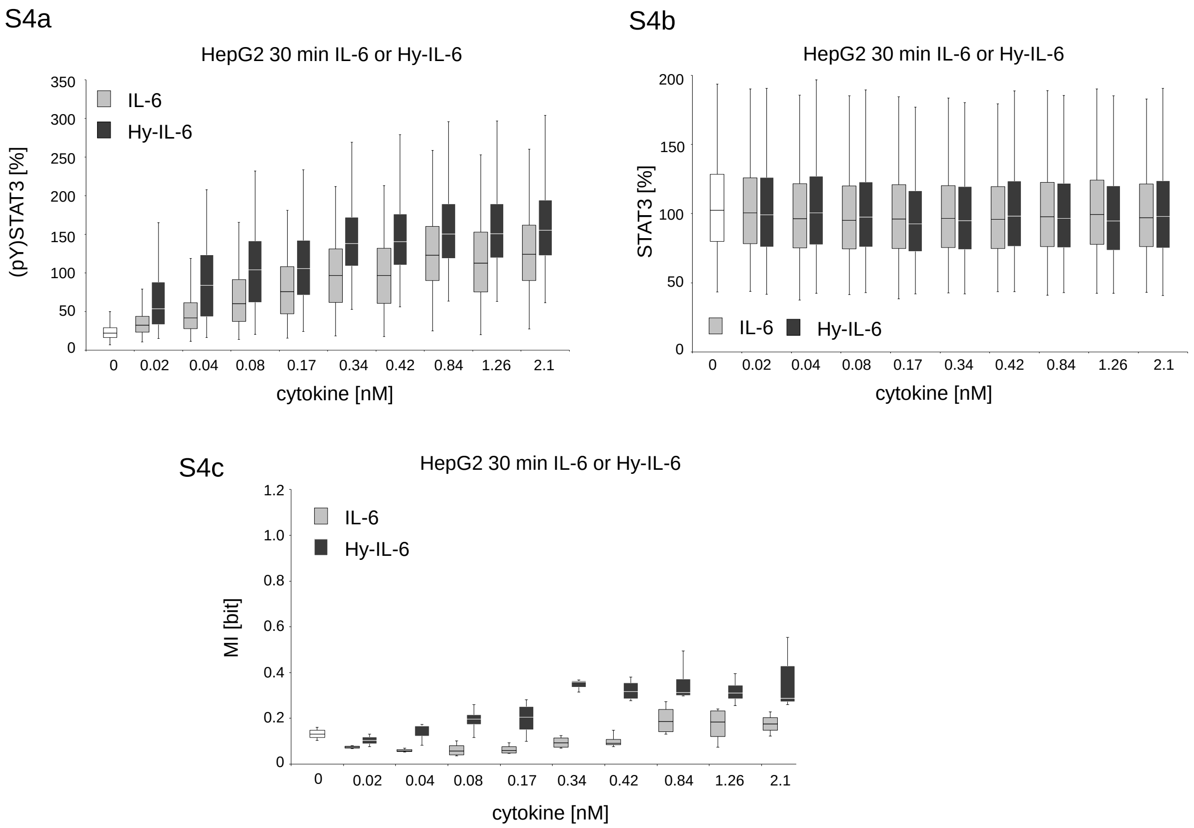

Supplementary Figure 4. Robustness of STAT3 phosphorylation decreases with the strength of stimulation in HepG2 cells

a, b) HepG2 cells were stimulated with increasing equimolar amounts of IL-6 or Hy-IL-6 respectively for 30 min. STAT3 expression and phosphorylation were evaluated by intracellular multiplex flow cytometry using specific fluorescent antibodies against STAT3 (p)Y705 (a) and STAT3 (b). For independent experiments mean fluorescence of cells per cytokine dose was calculated. Maximal fluorescence in each experiment was normalised to 100 %. Data are pooled from n = 4 experiments. **c)** Mutual Information between STAT3 expression and IL-6-induced STAT3 phosphorylation in HepG2 cells stimulated with IL-6 (light grey) or Hy-IL-6 (dark grey) for 30 min was calculated. Data from n = 4 independent experiments.

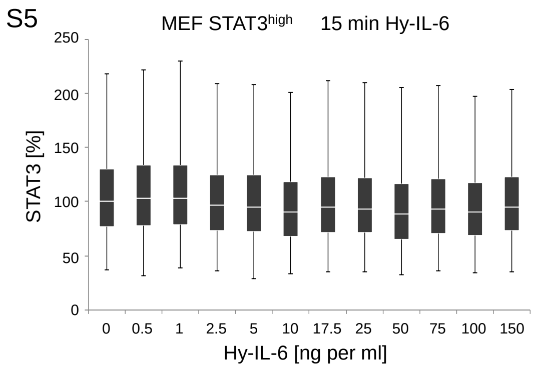

Supplementary Figure 5. STAT3 expression in MEF STAT3^{high} cells is not influenced by IL-6.

MEF STAT3^{high} cells were stimulated with increasing amount of Hy-IL-6 for 15 min. STAT3 phosphorylation and expression were evaluated by intracellular multiplex flow cytometry using specific fluorescent antibodies against STAT3 and STAT3 (p)Y705 (Fig. 3c). For independent experiments mean fluorescence of cells per cytokine dose was calculated. Maximal fluorescence in each experiment was normalised to 100 %. Data are pooled from n = 4 experiments.

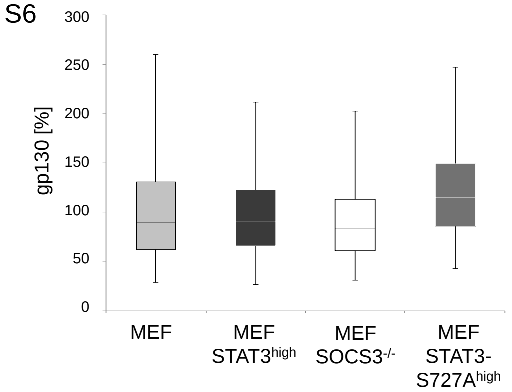

Supplementary Figure 6. Surface expression of glycoprotein 130 is comparable on MEF cell lines

10^6 MEF, MEF STAT3^{high}, MEF SOCS3$^{-/-}$, and MEF STAT-S727A^{high} cells were cultured on 6 cm dishes for 24 hours. Subsequently, cells were detached from cell-culture dishes with 1 ml Accutase (Biowest, Nuaillé, France Cat. No. L0950-100). Cells were washed once with BSA-EDTA-Buffer (2 % BSA, 2 mM EDTA in PBS) and stained overnight with a specific antibody against glycoprotein 130 (Mouse anti-gp130 (Clone BR-3), Hölzel Diagnostics, Cologne, Germany, Catalog No. 852.060.000 , Lot:P11016D6, 1:200). Subsequently cells were washed and stained with Anti-Mouse-PE antibody (Dianova, #115-116-146, 1:200) for 30 min. . Flow Cytometry Data were recorded on a BD FACS Canto II equipped with 3 lasers (405 nm, 488 nm, 663nm, Firmware Version 1.47) using FACS Diva (BD Biosciences), Version 6.1.3.Data were analysed using FlowJo (Treestar, Ashland, OR, USA), Version 10. Mean expression of glycoprotein 130 in MEF STAT3^{high} cells was normalised to 100 % in each experiment. Data are from n = 4 independent experiments.

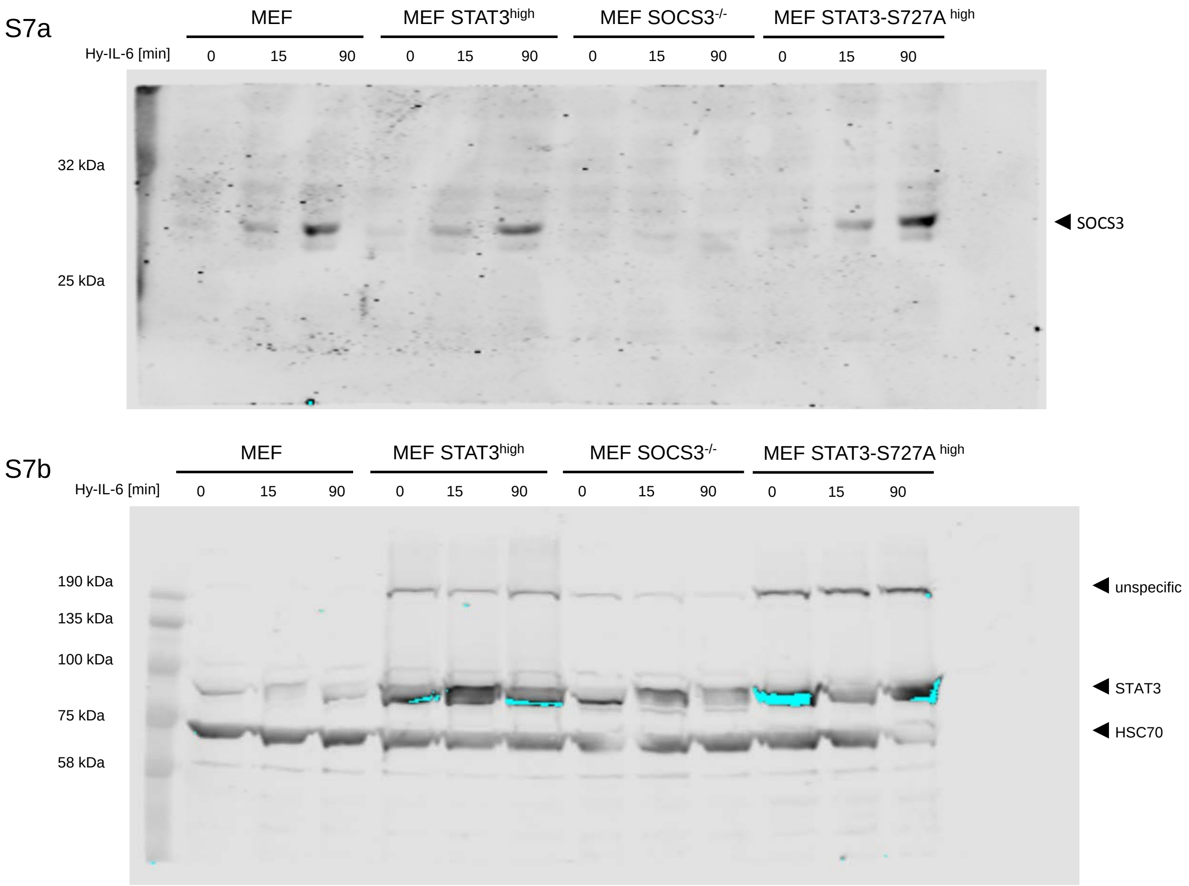

Supplementary Figure 7. Uncropped Western Blots for Fig. 4a.

a,b) MEF, MEF STAT3^{high}, MEF SOCS3$^{-/-}$, and MEF STAT3-S727A^{high} cells were stimulated with 75 ng Hy-IL-6 per ml for the indicated times. SOCS3 (a) and HSC70 (b) protein expression were evaluated by Western blotting. A representative result of n = 6 independent experiments is shown.

S8a

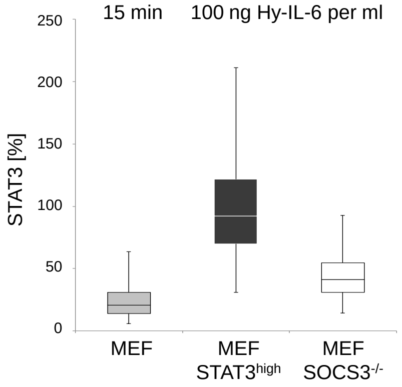

S8b

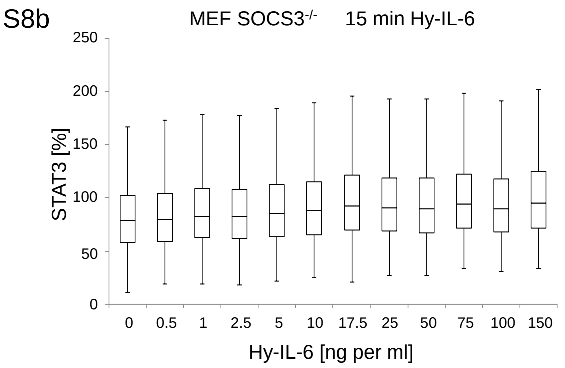

Supplementary Figure 8. STAT3 expression in MEF SOCS3$^{-/-}$ cells is not influenced by IL-6.

a) MEF, MEF STAT3^{high}, and MEF SOCS3$^{-/-}$ cells were stimulated with 100 ng Hy-IL-6 per ml for 15 min. Expression of STAT3 was analysed by intracellular flow cytometry. For independent experiments mean fluorescence of cells was calculated. Mean fluorescence of MEF STAT3^{high} cells was normalised to 100 %. Data are from n = 3 experiments. **b)** MEF SOCS3$^{-/-}$ cells were stimulated with increasing amount of Hy-IL-6 for 15 min. STAT3 phosphorylation and expression were evaluated by intracellular multiplex flow cytometry using specific fluorescent antibodies against STAT3 and STAT3 (p)Y705 (Fig. 4d). For independent experiments mean fluorescence of cells per cytokine dose was calculated. Maximal fluorescence in each experiment was normalised to 100 %. Data are pooled from n = 4 experiments.

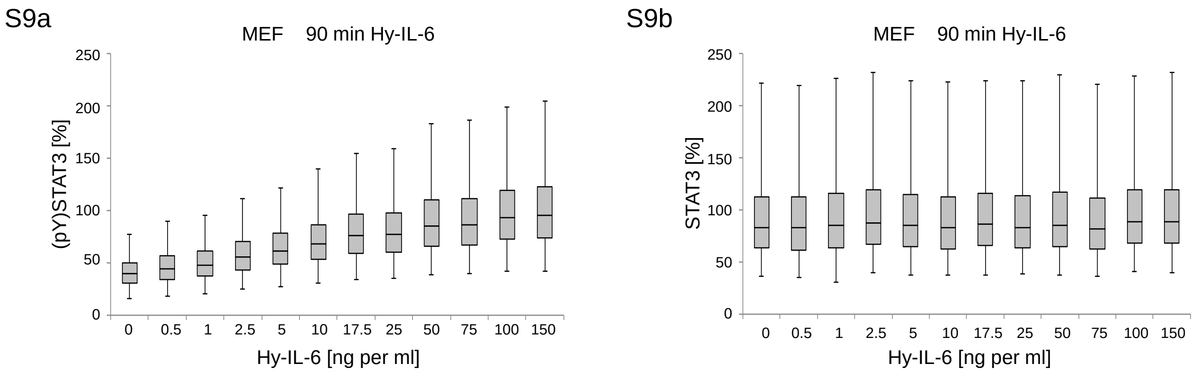

Supplementary Figure 9. STAT3 phosphorylation and expression in MEF cells stimulated for 90 min.

a, b) MEF cells were stimulated with increasing amount of Hy-IL-6 for 90 min. STAT3 phosphorylation and expression were evaluated by intracellular multiplex flow cytometry using specific fluorescent antibodies against STAT3 (p)Y705 (a) and STAT3 (b). For independent experiments mean fluorescence of cells per stimulus concentration was calculated. Maximal fluorescence in each experiment was normalised to 100 %. Data are pooled from n = 3 experiments.

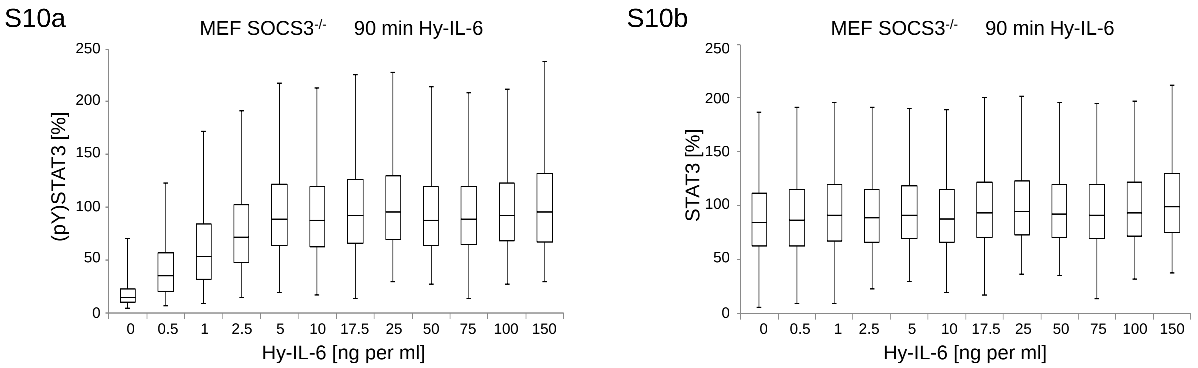

Supplementary Figure 10. STAT3 phosphorylation and expression in MEF SOCS3$^{-/-}$ cells stimulated for 90 min.

a, b) MEF SOCS3$^{-/-}$ cells were stimulated with increasing amount of Hy-IL-6 for 90 min. STAT3 phosphorylation and expression were evaluated by intracellular multiplex flow cytometry using specific fluorescent antibodies against STAT3 (p)Y705 (a) and STAT3 (b). For independent experiments mean fluorescence of cells per stimulus concentration was calculated. Maximal fluorescence in each experiment was normalised to 100 %. Data are pooled from n = 3 experiments.

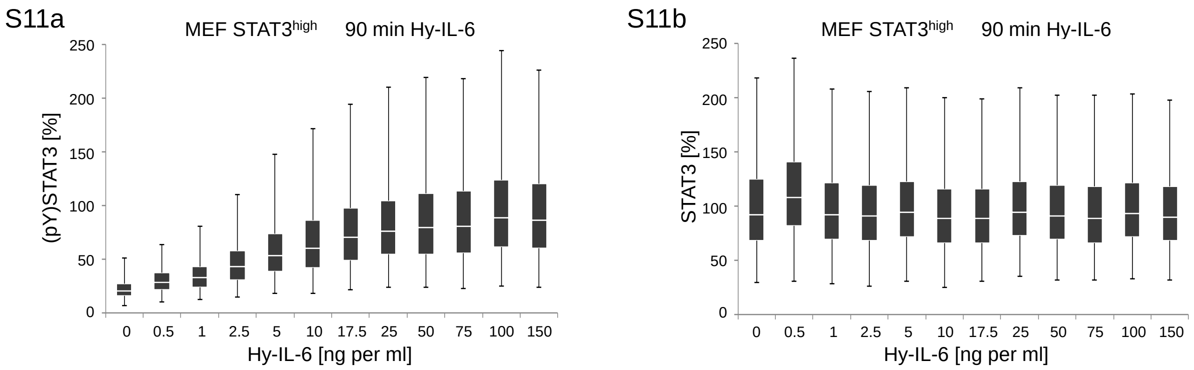

Supplementary Figure 11. STAT3 phosphorylation and expression in MEF STAT3^{high} cells stimulated for 90 min.

a,b) MEF STAT3^{high} cells were stimulated with increasing amount of Hy-IL-6 for 90 min. STAT3 phosphorylation and expression were evaluated by intracellular multiplex flow cytometry using specific fluorescent antibodies against STAT3 (p)Y705 (a) and STAT3 (b). For independent experiments mean fluorescence of cells per cytokine dose was calculated. Maximal fluorescence in each experiment was normalised to 100 %. Data are pooled from n = 4 experiments.

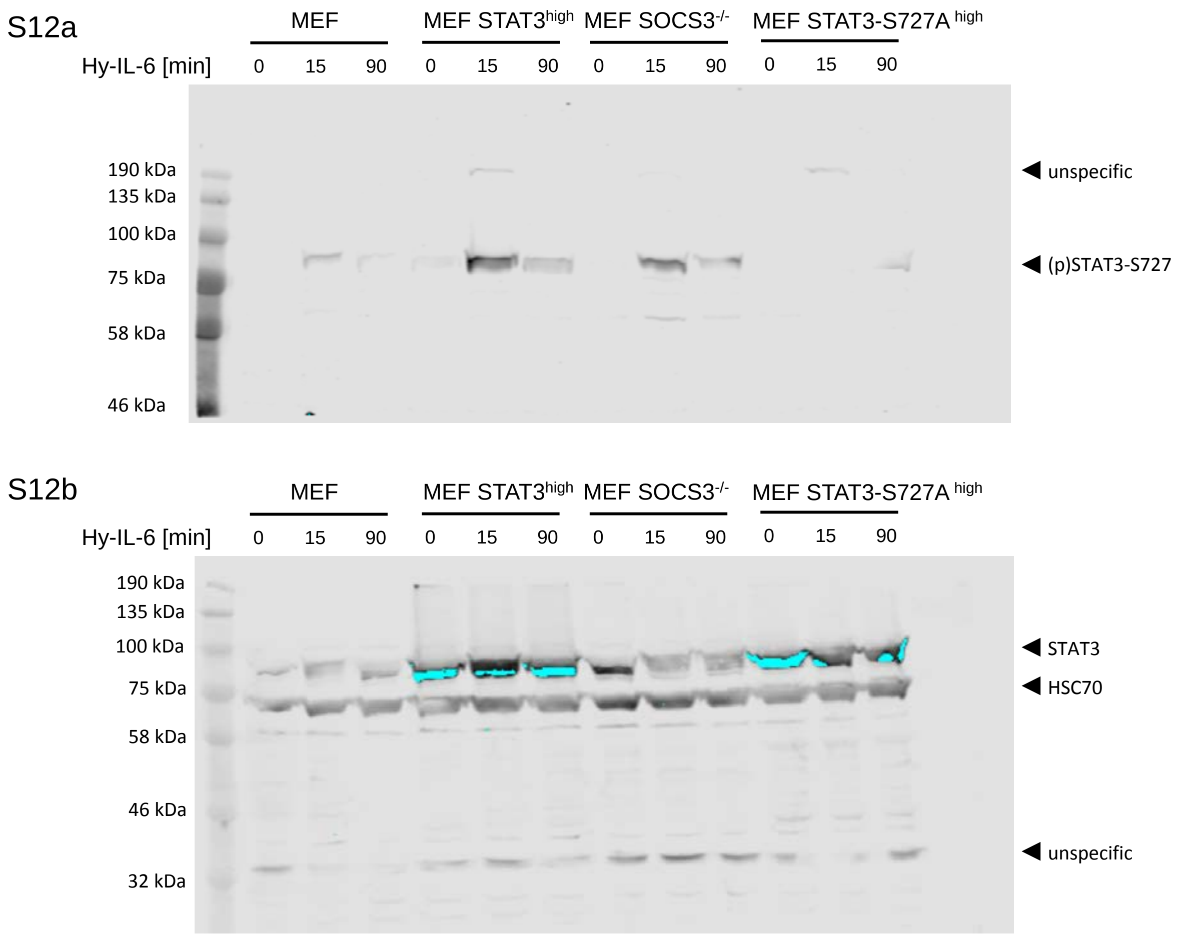

Supplementary Figure 12. Uncropped Western Blots for Fig. 5b.

a,b) MEF, MEF STAT3^{high}, MEF SOCS3$^{-/-}$, and MEF STAT3-S727A^{high} cells were stimulated with 75 ng Hy-IL-6 per ml for the indicated times. STAT3 S727 phosphorylation (a) and HSC70 (b) protein expression were evaluated by Western blotting. A representative result of n = 6 independent experiments is shown.

S13

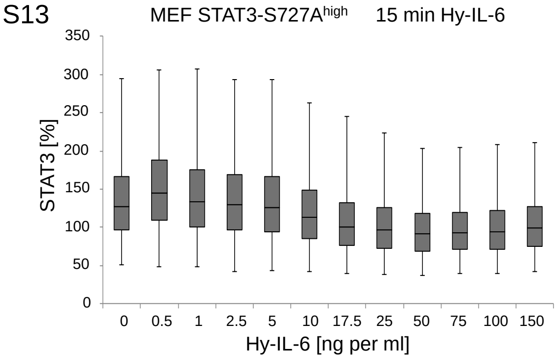

Supplementary Figure 13. STAT3 expression in MEF STAT3-S727A^{high} cells is not influenced by 15 min IL-6.

MEF STAT3-S727A^{high} cells were stimulated with increasing amount of Hy-IL-6 for 15 min. STAT3 expression and phosphorylation were evaluated by intracellular multiplex flow cytometry using specific fluorescent antibodies against STAT3 and STAT3 (p)Y705 (Fig. 5d). For independent experiments mean fluorescence of cells per cytokine dose was calculated. Maximal fluorescence in each experiment was normalised to 100 %. Data are pooled from n = 3 experiments.

S14

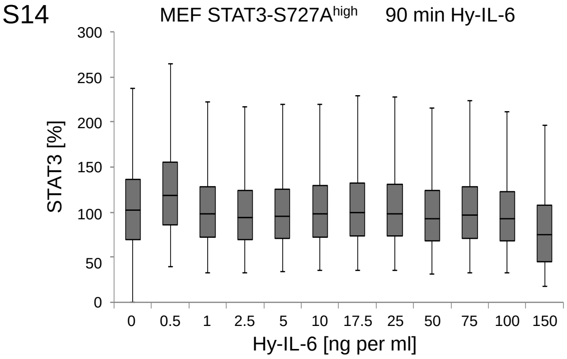

Supplementary Figure 14. STAT3 expression in MEF STAT3-S727A^{high} is not influenced by 90 min IL-6.

MEF STAT3-S727A^{high} cells were stimulated with increasing amount of Hy-IL-6 for 90 min. STAT3 expression and phosphorylation were evaluated by intracellular multiplex flow cytometry using specific fluorescent antibodies against STAT3 and STAT3 (p)Y705 (Fig. 5e). For independent experiments mean fluorescence of cells per cytokine dose was calculated. Maximal fluorescence in each experiment was normalised to 100 %. Data are pooled from n = 3 experiments.

Supplementary References

1. Kamentsky L*, et al.* Improved structure, function and compatibility for CellProfiler: modular high-throughput image analysis software. *Bioinformatics* **27**, 1179-1180 (2011).
